# The Positive Rhinovirus/Enterovirus Detection and SARS-CoV-2 Persistence beyond the Acute Infection Phase: An Intra-Household Surveillance Study

**DOI:** 10.3390/v13081598

**Published:** 2021-08-12

**Authors:** Pedro Brotons, Iolanda Jordan, Quique Bassat, Desiree Henares, Mariona Fernandez de Sevilla, Sara Ajanovic, Alba Redin, Vicky Fumado, Barbara Baro, Joana Claverol, Rosauro Varo, Daniel Cuadras, Jochen Hecht, Irene Barrabeig, Juan Jose Garcia-Garcia, Cristian Launes, Carmen Muñoz-Almagro

**Affiliations:** 1 Pediatric Infectious Diseases Research Group, Institut de Recerca Sant Joan de Déu, Esplugues de Llobregat, 08950 Barcelona, Spain; pedro.brotons@sjd.es (P.B.); yolanda.jordan@sjd.es (I.J.); desiree.henares@sjd.es (D.H.); mariona.fernandez@sjd.es (M.F.d.S.); alba.redin@sjd.es (A.R.); victoria.fumado@sjd.es (V.F.); juanjose.garciag@sjd.es (J.J.G.-G.); cristian.launes@sjd.es (C.L.); 2 Department of Medicine, School of Medicine, Universitat Internacional de Catalunya, Sant Cugat, 08195 Barcelona, Spain; 3 Consorcio de Investigacion Biomédica en Red Epidemiologia y Salud Pública (CIBERESP), 28029 Madrid, Spain; quique.bassat@isglobal.org (Q.B.); ibarrabeig@gencat.cat (I.B.); 4 Pediatric Intensive Care Unit, Hospital Sant Joan de Déu, Esplugues de Llobregat, 08950 Barcelona, Spain; 5 Pediatrics Department, Hospital Sant Joan de Déu, Esplugues de Llobregat, 08950 Barcelona, Spain; 6 Centro de Investigação em Saúde de Manhiça (CISM), Manhiça 1929, Mozambique; 7 ISGlobal, Hospital Clínic-Universitat de Barcelona, 08036 Barcelona, Spain; sara.ajanovic@isglobal.org (S.A.); barbara.baro@isglobal.org (B.B.); rosauro.varo@isglobal.org (R.V.); 8 Institució Catalana de Recerca i Estudis Avançats (ICREA), 08010 Barcelona, Spain; 9 Clinical Research Unit, Fundació Sant Joan de Déu, Esplugues de Llobregat, 08950 Barcelona, Spain; joana.claverol@sjd.es (J.C.); daniel.cuadras@sjd.es (D.C.); 10 Centre for Genomic Regulation (CRG), Genomics Unit, 08003 Barcelona, Spain; jochen.hecht@crg.eu; 11 Epidemiological Surveillance Unit, Department of Health, Generalitat de Catalunya, 08907 Barcelona, Spain; 12 Department of Molecular Epidemiology, Hospital Sant Joan de Déu, Esplugues de Llobregat, 08950 Barcelona, Spain

**Keywords:** SARS-CoV-2, COVID-19, RT-PCR, rhinovirus, household

## Abstract

We aimed to assess the duration of nasopharyngeal severe acute respiratory syndrome coronavirus 2 (SARS-CoV-2) RNA persistence in adults self-confined at home after acute infection; and to identify the associations of SARS-CoV-2 persistence with respiratory virus co-detection and infection transmission. A cross-sectional intra-household study was conducted in metropolitan Barcelona (Spain) during the time period of April to June 2020. Every adult who was the first family member reported as SARS-CoV-2-positive by reverse transcription polymerase chain reaction (RT-PCR) as well as their household child contacts had nasopharyngeal swabs tested by a targeted SARS-CoV-2 RT-PCR and a multiplex viral respiratory panel after a 15 day minimum time lag. Four-hundred and four households (404 adults and 708 children) were enrolled. SARS-CoV-2 RNA was detected in 137 (33.9%) adults and 84 (11.9%) children. Rhinovirus/Enterovirus (RV/EV) was commonly found (83.3%) in co-infection with SARS-CoV-2 in adults. The mean duration of SARS-CoV-2 RNA presence in adults’ nasopharynx was 52 days (range 26–83 days). The persistence of SARS-CoV-2 was significantly associated with RV/EV co-infection (adjusted odds ratio (aOR) 9.31; 95% CI 2.57–33.80) and SARS-CoV-2 detection in child contacts (aOR 2.08; 95% CI 1.24–3.51). Prolonged nasopharyngeal SARS-CoV-2 RNA persistence beyond the acute infection phase was frequent in adults quarantined at home during the first epidemic wave; which was associated with RV/EV co-infection and could enhance intra-household infection transmission.

## 1. Introduction

The infectious coronavirus disease 2019 (COVID-19) caused by the new severe acute respiratory syndrome coronavirus 2 (SARS-CoV-2) has had devastating public health consequences globally since it first emerged in Wuhan, China in December 2019 [1]. Spain was one of the most strongly affected countries, with over 3.7 million cases and around 80,000 of deaths as of June 2021 [2]. A state of emergency was declared by the national government in the period from March to May 2020, enforcing strict confinement for the vast majority of the population and the closure of most businesses and all leisure, cultural and educational places [3].

SARS-CoV-2 RNA detection by reverse transcription polymerase chain reaction (RT-PCR) is an accurate method that is widely used to diagnose and monitor COVID-19 [4]. Diverse case series studies have reported that SARS-CoV-2 RNA remains detectable in upper respiratory samples for between 11 and 20 days since symptom onset [5,6]. The impact of virus persistence on disease transmissibility remains incompletely understood [7]. 

The prevalence of SARS-CoV-2 co-infection with other respiratory viruses varies greatly in adults, from less than 5% [8,9,10] to up to 20% [11], whereas a co-infection rate of 40% has been reported in children [12]. It is unclear whether these co-infections lead to worse clinical outcomes and to what extent they are related with lower or higher SARS-CoV2 transmission [13]. Of note, hardly distinguishable rhinoviruses and respiratory enteroviruses (RVs/EVs), which are the main etiological agents of the common cold, often predominate over other respiratory viruses in patients infected with SARS-CoV-2 [8,9,10,11,12,13,14]. While an antagonistic interaction of RV/EV with SARS-CoV-2 has previously been described [10], the co-detection of both viral species has also been associated with mild COVID-19 [15].

A recent intra-household study conducted by our group during the spring 2020 lockdown reported that the SARS-CoV-2 seroprevalence in the child contacts of self-confined parents who had experienced COVID-19 was 17.6% [16]. In this nested study, we aimed to determine the duration of nasopharyngeal SARS-CoV-2 RNA presence in adults self-confined at home beyond the acute infection phase, and to assess the association between SARS-CoV-2 RNA persistence, respiratory virus co-detection and intra-household infection transmission. We also analyzed the epidemiological, clinical and microbiological factors related to SARS-CoV-2 RNA persistence.

## 2. Materials and Methods

### 2.1. Study Design

The study was conducted by researchers of the University Hospital Sant Joan de Déu (Barcelona, Spain). Details of participants and data and sample collection procedures have been previously described [16]. In brief, we prospectively enrolled volunteer families that included one adult parent who was the first family member reported as positive by SARS-CoV-2 RT-PCR (hereafter referred to as first-reported adult case) and at least one child aged less than 15 years co-habiting in the same household. Enrollment targeted family households located in the Health Region of metropolitan Barcelona and spanned from 28 April to 3 June 2020. Participant adults recovering or completely recovered from COVID-19 had tested positive for SARS-CoV-2 RNA detection in a nasopharyngeal swab at least 15 days before their household was visited for sample collection. Virus persistence in adults was defined as positive detection of SARS-CoV-2 RNA in a nasopharyngeal swab 15 days or longer after the first confirmatory RT-PCR result. All family members were also tested by rapid immunochromatographic lateral flow assay (LFA) at the household visit, whereas only adults that gave their consent had venous blood extracted for subsequent enzyme-linked immunosorbent assay (ELISA) at the laboratory of the study site.

### 2.2. Detection of SARS-CoV-2 RNA and RNA/DNA of Other Respiratory Viruses

SARS-CoV-2 RNA was extracted from nasopharyngeal swabs using the Quick-DNA/RNA Viral Mag Bead kit (Zymo Research, Irvine, CA, US) which was fully automated on a Tecan Dream Prep NAP Workstation (Tecan Trading AG, Männendorf, Switzerland). SARS-CoV-2 RT-PCR assays were performed in an Applied Biosystems 7900HT robot with 384-well block equipment according to the CDC-006-00019 protocol (virus RNA extraction and amplification procedures detailed in Appendix A). A SARS-CoV2 RNA result was considered positive if the RNase P human gene and either the N1 or N2 gene were detected, and negative if only Rnase P gene was detected. Those households where first-reported adult cases yielded invalid results (all three genes negative) by RT-PCR were excluded from the study.

Respiratory viruses other than SARS-CoV-2 were identified by Allplex^®^ Respiratory Panels 1, 2 and 3 (Seegene Inc., Seoul, Korea) according to manufacturer instructions. This real-time one-step RT-PCR panel assay targets adenovirus (AdV), bocavirus (BoV) types 1/2/3/4, coronavirus (CoV) types 229E/NL63/OC43, influenza A virus (IFV-A) including differentiation of subtypes H1/H1N1-2009/H3, influenza B virus (IFV-B), metapneumovirus (MPV), parainfluenza virus (PIV) types 1/2/3/4, respiratory syncytial virus (RSV) types A/B and rhinovirus/enterovirus (RV/EV).

### 2.3. Detection of SARS-CoV-2 Antibodies

A rapid immunochromatographic lateral flow assay (2019-n-CoV Ab Test, Innovita Tangshan Biological Technology Co, Beijing, China) was used for the detection of IgG, IgM, or both in capillary blood obtained by finger prick from all participants. In addition, serum samples from a number of first-reported adult cases were tested by the Abbott SARS-CoV-2 IgG assay on the Abbott Architect instrument according to the manufacturer’s instructions. This assay is a chemiluminescent immunoassay for the qualitative detection of IgG in human serum or plasma against the SARS-CoV-2 nucleoprotein.

### 2.4. Statistical Analysis

Dichotomous variables were compared by chi-square or Fisher’s exact test. The t-test or the Mann–Whitney test were respectively used for the comparison of continuous variables with normal distributions or skewed data. SARS-CoV-2 nasopharyngeal load was log transformed before analysis. Bivariate analyses were performed to identify the associations of the presence of SARS-CoV-2 RNA in the nasopharynx of participants with concomitant infection with other respiratory viruses, among other relevant epidemiological, microbiological and clinical data. Variables that showed a relationship with nasopharyngeal SARS-CoV-2 RNA detection at a *p*-value ≤ 0.10 were considered for multivariate logistic regression analysis. Statistical significance was set at a *p*-value of < 0.05 and 95% confidence intervals (CIs). Stata v.15 software (StataCorp, College Station, TX, US) was used for statistical analyses.

### 2.5. Ethics Statement

Every adult household member recruited for the main household study gave an informed consent to participate. Informed consent was also obtained from parents/guardians of participating children, as was individual assent from every participating child aged ≥12 years. The Ethics Committee of University Hospital Sant Joan de Déu approved the main household seroprevalence study, including the use of collected samples for further studies nested within it.

## 3. Results

### 3.1. Selection of Family Households

Four-hundred and ten families that participated in the main household seroprevalence study were screened, and six of them were excluded due to invalid results by SARS-CoV-2 RT-PCR for either the first-reported adult case or their children. A total of 404 family households were ultimately selected, including 404 first-reported adult cases and 708 child contacts (total participants, 1112).

### 3.2. Epidemiological and Clinical Characteristics of Participants

The majority of adults were women (*n* = 254, 62.9%) and health workers (*n* = 224, 55.4%). Adults’ mean age was 40.3 years (SD 7.1), and women were younger (mean age 40.1 years, SD 7.2) than men (mean age 43.5 years, SD 6.3, *p* < 0.001). The boys predominated among child contacts (*n* = 379, 53.5%), and were younger (mean age 5.6 years, SD 3.7) than girls (mean age 6.4 years, SD 3.8, *p* = 0.01). Household mean surface area was 102 square meters (SD 43.0) and co-habitants ranged from 2 to 7 members, including 1 to 5 children. There was more than one child living in 311 (74.5%) households. Twenty-nine (7.2%) households were visited within 18–30 days after the first positive SARS-CoV-2 RT-PCR result for the first-reported adult case, 253 (62.6%) within 31–60 days and 122 (30.2%) beyond 60 days. Mean time lag between initial and follow-up SARS-CoV-2 RT-PCR tests in cases was 52 days (range 18–85 days). Overall, 93 (23.1%) out of 404 adults were hospitalized during the acute phase of infection and men had a higher hospitalization rate than women (39.3 vs. 13.4%, *p* < 0.001). Co-morbidities were reported in 27.2% of cases, particularly obesity (12.5%) and autoimmune diseases (7.1%). Nearly all children (99.0%) had been vaccinated according to the pediatric vaccination schedule applicable in the region. A noticeable proportion of children (35.9%) had experienced respiratory infections since January 2020 (Table 1).

### 3.3. SARS-CoV-2 RNA Detection and Antibody Response in Participants

Persistence of SARS-CoV2 RNA detection was observed in the nasopharynx of 137 (33.9%) adults. The mean duration of SARS-CoV-2 RNA persistence in adults was 52 days (range 26–83 days). Mean nasopharyngeal viral load was 3.4 log copies/mL (SD, 1.0). We found 180 (44.6%) out of 404 adults to be positive by SARS-CoV-2 antibody LFA and 214 (85.6%) out of 250 positive by ELISA. Among the 137 adults persistently positive for the virus according to RT-PCR, 73 (53.3%) had a negative LFA result whereas 10 (11.1%) out of 90 tested by ELISA were negative. The difference in mean viral load between LFA-negative and LFA-positive adults was not significant (3.5 vs. 3.3 log copies/mL, *p* = 0.36). A trend for a significantly higher mean viral load was identified between those that were ELISA-negative (4.0 log copies/mL) and positive (3.4 log copies/mL, *p* = 0.05). The mean duration of nasopharyngeal SARS-CoV-2 RNA persistence among first-reported adult cases with no detectable antibody response was very similar to that obtained for all cases, either using LFA (53 days, range 26–83 days) or ELISA (56 days, range 31–71 days).

Nasopharyngeal SARS-CoV-2 RNA was detected in 84 (11.9%) child contacts, but almost all of those found positive (99.9%) were paucisymptomatic or asymptomatic, except for one girl who was hospitalized due to multi-systemic Kawasaki-like inflammatory syndrome. Mean nasopharyngeal viral load was 3.3 log copies/mL (SD, 0.9). Among the 84 children with detectable nasopharyngeal SARS-CoV-2 RNA, 40 (47.6%) were identified as negative by LFA. LFA-positive children had considerably higher viral density in their nasopharynx than those that were LFA-negative (3.6 vs. 2.9 log copies/mL, *p* = 0.001).

### 3.4. Nasopharyngeal Detection of Other Respiratory Viruses in Participants

**Figure 1:**
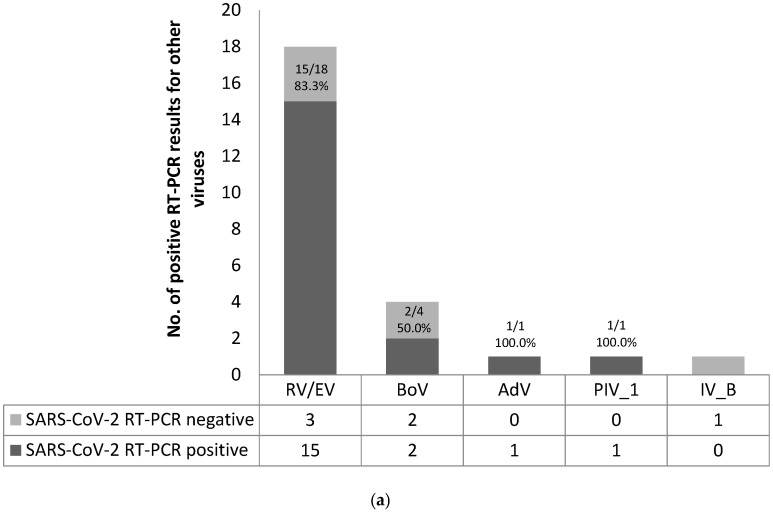

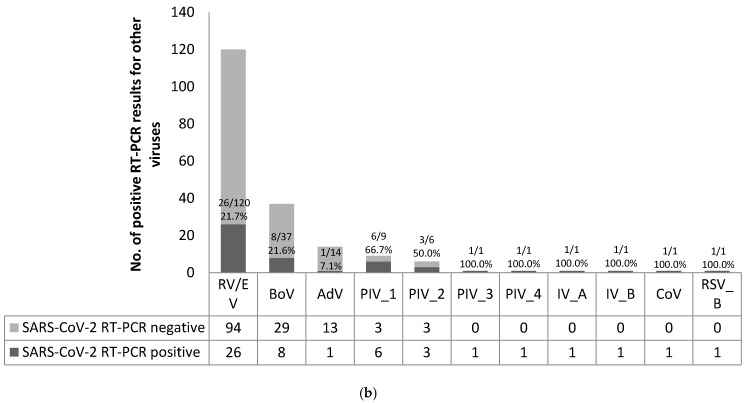
Distribution of other respiratory viruses: (**a**) in first-reported adult cases; (**b**) in children. Abbreviations: SARS-CoV-2, severe acute respiratory syndrome coronavirus 2; RT-PCR, reverse-transcriptase polymerase chain reaction; RV/EV, rhinovirus/enterovirus; BoV, bocavirus; AdV, adenovirus; PIV_1/2/3/4, parainfluenza virus 1/2/3/4; IV A/B, influenza virus type A/B; CoV, coronavirus; RSV_B, respiratory syncytial virus type B.

A valid result of the multiplex RT-PCR performed for the screening of 16 respiratory viruses was obtained in 403 first-reported adult cases and 707 children. Respiratory viruses other than SARS-CoV-2 were identified in 20 (5.0%) adults and 157 (22.2%) children. RV/EV was most frequently detected among adults (*n* = 18, 90.0%) and was commonly found in co-infection with SARS-CoV-2 (*n* = 15, 83.3%). RV/EV also predominated in children (*n* = 120, 76.4%), but co-infection with SARS-CoV-2 (*n* = 26, 21.7%) was less frequent (Figure 1).

### 3.5. Persistent Nasopharyngeal SARS-CoV-2 RNA Detection in First-Reported Adult Cases and Associated Factors

**Figure 2:**
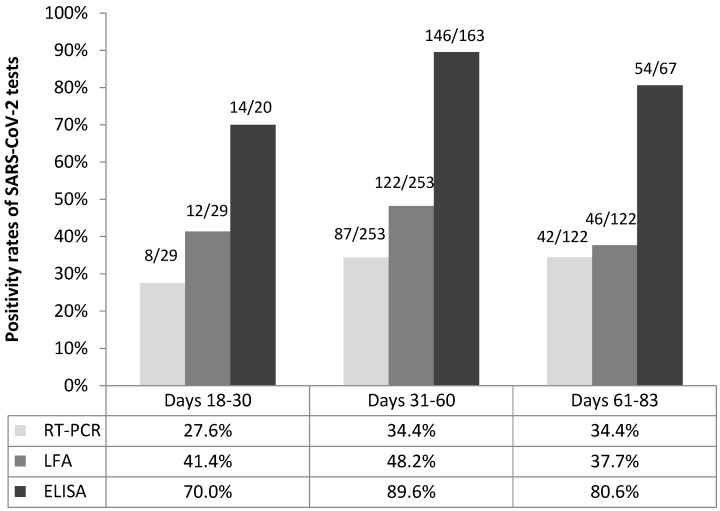
SARS-CoV-2 RT-PCR and antibody positivity rates according to time elapsed since first positive SARS-CoV-2 RT-PCR result in first-reported adult cases. Abbreviations: SARS-CoV-2, severe acute respiratory syndrome coronavirus 2; RT-PCR, reverse-transcriptase polymerase chain reaction; LFA, immunochromatographic lateral flow assay; ELISA, enzyme-linked immunosorbent assay.

A markedly higher proportion of SARS-CoV-2 RNA presence in the nasopharynx was observed in health workers when compared to other professionals (39.3 vs. 24.8%, *p* = 0.004) but mean viral density was identical in the two groups (3.4 log copies/mL, *p* = 0.75). We identified a trend for statistical significance in the rate of nasopharyngeal viral persistence in women compared with men (37.4 vs. 28.0%, *p* = 0.05) even though mean viral load was similar in females (3.4 log copies/mL) and males (3.3 log copies/mL, *p* = 0.70). Prolonged SARS-CoV-2 detection was more frequent in adult cases that had not been hospitalized in the previous COVID-19 episode than in those that had received hospital care (36.5 vs. 25.8%, *p* = 0.06). However, mean SARS-CoV-2 RNA load was the same among former outpatients and inpatients (3.4 log10 copies/mL, *p* = 0.84). Minor differences in the proportions of nasopharyngeal viral persistence were found according to the time lag elapsed between initial and follow up RT-PCR tests: 8/29 (27.6%) among parents re-tested within 18–30 days, 87/253 (34.4%) in those re-tested within 31–60 days and 42/122 (34.4%) in those re-tested after 60 days (*p* = 0.76) (Figure 2). 

Comparable rates of nasopharyngeal SARS-CoV-2 RNA persistence were observed according to results of LFA: 64/180 (35.6%) in LFA-positive adults and 73/224 (32.6%) in those that were negative (*p* = 0.53). Additionally, adults with available ELISA results showed similar viral persistence rates regardless of positive (80/214, 37.4%) or negative (10/36, 27.8%) results by that test (*p* = 0.27). There was a significant association between the persistent detection of SARS-CoV-2 RNA in the nasopharynx of first-reported adult cases and the detection of RV/EV: 15 (83.3%) out of 18 individuals with RV/EV infection showed prolonged SARS-CoV-2 RNA detection in comparison with 122 (31.7%) out of 385 without RV/EV infection (*p* < 0.001). Nonetheless, mean SARS-CoV-2 viral load was similar in co-infected adults (3.7 log copies/mL) and in those not co-infected (3.4 log copies/mL, *p* = 0.22). In multivariate regression, co-infection with RV/EV (adjusted odds ratio (aOR) 9.31, 95% CI 2.57–33.80) and being a health worker (aOR 1.75, 95% CI 1.04–2.94) were found to be independent risk factors related with SARS-CoV-2 RNA persistence (Table 2).

### 3.6. Nasopharyngeal SARS-CoV-2 RNA Detection in Children According to Virus Persistence in Adults and Other Factors

A significantly higher RT-PCR positivity rate was observed among children living with a first-reported adult case who had persistent SARS-CoV-2 infection (48/234, 20.5%) than in those whose parent did not show virus persistence (36/474, 7.6%, *p* = 0.001). There were also remarkable differences in the virus detection rate between children positive by LFA (44/122, 36.1%) compared with those that were LFA-negative (40/586, 6.8%, *p* < 0.001). Specifically, the proportion of children positive by SARS-CoV-2 RT-PCR who co-habited with adults showing prolonged virus RNA detection and no detectable antibody response by LFA (24/128, 18.8%) was substantially higher than that of RT-PCR-positive children whose parents did not show persistent virus RNA detection (9/255, 3.5%, *p* = 0.001).

The mean nasopharyngeal viral load in adults persistently positive by SARS-CoV-2 RT-PCR and with no antibody response by LFA displayed no significant influence on the presence or absence of SARS-CoV-2 RNA in children (3.7 vs. 3.5 log copies/mL, *p* = 0.43). On the other hand, mean nasopharyngeal SARS-CoV-2 RNA load in children co-infected by RV/EV was significantly higher than in those not co-infected (3.8 vs. 3.0 log copies/mL, *p* < 0.001). However, SARS-CoV-2 detection among children was not related with the presence or absence of RV/EV infection in adults (20.6 vs. 11.5%, *p* = 0.11). A multivariate regression model revealed that SARS-CoV-2 antibody detection by LFA (aOR 7.20, 95% CI 4.27–12.15), persistent nasopharyngeal viral RNA presence in first-reported adult cases (aOR 2.08, 95% CI 1.24–3.51) and RV/EV infection in children (aOR 2.04, 95% CI 1.13–3.68) were correlated with SARS-CoV-2 RNA detection in children (Table 3).

## 4. Discussion

The main findings of this study were the frequent and long persistence of SARS-CoV-2 RNA in the nasopharynx of first-reported adult cases recovering or recovered from COVID-19 and the correlation of virus persistence in adults with RV/EV co-infection and SARS-CoV-2 RNA detection in their household child contacts. Our results showed that nasopharyngeal SARS-CoV-2 RNA can remain detectable in the post-acute infection phase for an extended period of time, even in the absence of antibody response assessed either by LFA or ELISA. The observed duration of SARS-CoV-2 persistence in adults’ nasopharynx was far longer than the previously described period of up to 37 days [17]. The proportion of adults with active infection, as measured by a negative ELISA result, was within the rates of SARS-CoV-2 RNA positivity ranging from 6.4 to 21.4% that were observed in patients who were convalescent or had recovered from COVID-19, either during their stay at hospital [18] or once discharged home [19]. Our hypothesis is that the lack of results reporting prolonged nasopharyngeal SARS-CoV-2 RNA detection similar to ours may be partly attributable to the difficulty of conducting post-infection observations on COVID-19 patients discharged home or managed as outpatients for long periods of time.

The nasopharyngeal SARS-CoV-2 RNA load of first-reported adult cases was relatively low, probably due to their post-acute infection status, and we did not find substantial differences in viral load between adults co-habiting with infected children or with children free from infection, probably due to the post-acute infection status of cases. Diverse studies have not been able to recover viable SARS-CoV-2 from convalescent patients persistently positive by RT-PCR beyond the second week after symptom onset [20,21]. Low viral loads measured in respiratory samples of COVID-19 acute patients have also been shown to have reduced infectivity in vitro [22,23,24]. Based on the remarkable rate of positive children living with a parent showing SARS-CoV-2 RNA persistence, we speculate that virus transmission occurred in a noticeable number of households as a consequence of prolonged exposure of contacts to infected cases in enclosed settings such as households rather than being enhanced by cases with high viral load. Interestingly, the mean size of households enrolled in the study apparently enabled the maintenance of social distancing between cases and contacts, which highlights the importance of reinforcing awareness of adherence to preventive measures in quarantined households, including mask wearing, frequent hand washing and adequate indoor air ventilation practices.

RV/EV appeared as important viral species co-detected in the nasopharynx of both adults and children positive for SARS-CoV-2 by RT-PCR. The role of RV in SARS-CoV-2 acquisition and transmission is open to debate. Since RV has been described to have a faster growth rate than SARS-CoV-2, it has been suggested that RV may mitigate SARS-CoV-2 infection initiated some time before or suppress it if co-infection occurs simultaneously [13]. In this regard, the subsequent acquisition of a respiratory pathogen by patients already infected by SARS-CoV-2 has been reported to be a frequent sequential pattern of co-infection [15]. Angiotensin-converting enzyme 2 (ACE2) may play a crucial factor in the interaction between SARS-CoV-2 and RV. ACE2 has recently been acknowledged to be crucial for the replication of SARS-CoV-2 [25]. In addition, the upregulation of ACE2 in response to RV serotype A16, responsible for the majority of RV infections in children and young adults, was recently demonstrated in an ex vivo experimental study using epithelial cells from children [26]. Other experimental studies of the original SARS coronavirus in mice have postulated that the upregulation of ACE2 expression in response to RV infection could facilitate SARS-CoV-2 acquisition and transmission, as well as modulate disease severity [27,28]. The frequent SARS-CoV-2 and RV/EV co-detection in the nasopharynx of first-reported adults and the common presence of SARS-CoV-2 in child contacts might be indicative of the potential mechanism of RV infection for increased ACE2 expression facilitating SARS-CoV-2 acquisition by adults and transmission to their child contacts.

The principal strength of this study was the use of a large sample size of family households and the conduct of the study under strict quarantine conditions, which made it possible to rule out any biases produced by social interactions of family members outside of the home. The findings reported are subject to some limitations. First, given the cross-sectional nature of our study, we were unable to evaluate both the direction of transmission between first-reported adult cases and children and the timing of SARS-CoV-2 and RV/EV infection in adults and children. Although the mean time lag between adults’ first and second positive RT-PCR tests was 52 days (an interval far longer than the mean incubation time of about 4–6 days reported for SARS-CoV-2 infection [29]), the possibility that a number of child contacts could have become infected even before their parent, remaining asymptomatic until being tested, cannot be completely discarded. Secondly, we cannot extrapolate results to quarantined households exhibiting demographic or occupancy patterns different from those reported in this study. Thirdly, the multiplex RT-PCR test used for the detection of 16 respiratory viruses did not make any distinction between genetically similar RV and EV.

## 5. Conclusions

Prolonged nasopharyngeal SARS-CoV-2 RNA persistence beyond the acute infection phase was frequent among adults self-confined at home during the first epidemic wave, was associated with RV/EV co-infection and could enhance intra-household infection transmission.

## Figures and Tables

**Table 1:** Epidemiological and clinical characteristics of participants.

Variable	No. (%)
First-reported adult cases	404 (100.0)
Mean age (SD), years	41.3 (7.1)
15–24	1 (0.3)
25–34	47 (11.6)
35–44	261 (64.6)
45–55	89 (22.0)
≥55	6 (1.5)
Sex, female	254 (62.9)
Health worker	224 (55.4)
Obesity ^a^ (*n* = 401)	50 (12.5)
Autoimmune disease (*n* = 379)	27 (7.1)
Asthma (*n* = 379)	19 (5.0)
Hypertension ^b^ (*n* = 402)	14 (3.5)
Hospitalization during the past COVID-19 episode	93 (23.0)
Child contacts	708 (100.0)
Mean age (SD), years	6.0 (3.8)
0–4	344 (48.6)
5–14	364 (51.4)
Sex, male	379 (53.5)
Vaginal delivery (*n* = 695)	488 (70.2)
Breastfeeding (*n* = 693)	594 (85.7)
Vaccines received according to vaccination schedule	689 (99.0)
Recent respiratory infection ^c^	254 (35.9)
Attendance of kindergarten/school before lockdown (*n* = 702)	196 (27.9)
Public transport use to go to school before lockdown (*n* = 693)	97 (14.0)

Values expressed as No. (%), unless otherwise stated. Abbreviations: SD, standard deviation; COVID-19, coronavirus disease 2019. ^a^ Obesity defined as a body mass index ≤ 30.0. ^b^ Hypertension defined as systolic blood pressure readings ≥ 120 mm Hg and diastolic blood pressure readings < 80 mm Hg. ^c^ Since January 2020.

**Table 2:** Factors associated with persistent nasopharyngeal SARS-CoV-2 RNA detection in first-reported adult cases.

Variable	Group 1 ^a,b^	Group2 ^a,b^	Univariate Analysis		Multivariate Analysis	
	SARS-CoV-2 Positive ^c^ (%)	SARS-CoV-2 Positive ^c^ (%)	OR (95% CI)	*p*-Value	aOR (95% CI)	*p*-Value
Age, 15–40 vs. >40 years	65/188 (34.6)	72/216 (33.3)	0.95 (0.63–1.43)	0.79		
Sex, female vs. male	95/254 (37.4)	42/150 (28.0)	1.54 (0.99–2.38)	0.05	1,44 (0.87–2.39)	0.16
Profession, health worker vs. others	88/224 (39.3)	36/145 (24.8)	1.96 (1.23–3.11)	0.004	1.75 (1.04–2.94)	0.04
Obesity, yes vs. no	18/50 (36.0)	118/351 (33.6)	1.11 (0.60–2.06)	0.74		
Hypertension, yes vs. no	10/27 (37.0)	117/352 (33.2)	1.18 (0.52–2.66)	0.69		
COVID-19 severity, hospital care vs. outpatient care	24/93 (25.8)	113/311 (36.3)	0.61 (0.36–1.02)	0.06	0.97 (0.53–1.79)	0.93
Rhinovirus/enterovirus infection, yes vs. no	15/18 (83.3)	122/385 (31.7)	10.78 (3.06–37.93)	<0.001	9.31 (2.57–33.80)	0.001
Antibody detection by LFA, yes vs. no	64/180 (35.6)	73/224 (32.6)	1.14 (0.75–1.73)	0.53		
Antibody detection by ELISA, yes vs. no	80/214 (37.4)	10/36 (27.8)	1.55 (0.71–3.39)	0.27		

Abbreviations: SARS-CoV-2, severe acute respiratory syndrome coronavirus 2; OR, odds ratio; aOR, adjusted odds ratio; CI, confidence interval; COVID-19, coronavirus disease 2019; LFA, immunochromatographic lateral flow assay; ELISA, enzyme-linked immunosorbent assay. ^a^ Group 1 refers to the variable group mentioned in the first place (i.e., health worker) and Group 2 refers to the variable group mentioned in the second place (i.e., other professions). ^b^ ≥10 events of interest per variable. ^c^ Positive by the follow-up SARS-CoV-2 RT-PCR test (indicator of virus persistence).

**Table 3:** Factors associated with persistent nasopharyngeal SARS-CoV-2 RNA detection in first-reported adult cases.

Variable	Group 1 ^a,b^	Group2 ^a,b^	Univariate Analysis		Multivariate Analysis	
	SARS-CoV-2 Positive ^c^ (%)	SARS-CoV-2 Positive ^c^ (%)	OR (95% CI)	*p*-Value	aOR (95% CI)	*p*-Value
Age, 5–14 vs. 0–4 years	46/364 (12.6)	38/344 (11.1)	1.08 (0.86–1.36)	0.51		
Sex, female vs. male	35/329 (10.6)	49/379 (12.9)	0.80 (0.50–1.27)	0.35		
Delivery type, C-section vs. vaginal	23/207 (11.1)	60/488 (12.3)	0.89 (0.54–1.49)	0.66		
Feeding type, breastfeeding vs. formula	68/594 (11.5)	15/99 (15.2)	0.72 (0.40–1.33)	0.29		
Vaccination according to schedule, yes vs. no	83/689 (12.1)	0/7 (0.0)	1.00	0.33		
Recent respiratory infection ^c^, yes vs. no	31/254 (12.2)	53/452 (11.7)	1.05 (0.65–1.68)	0.85		
Kindergarten/school attendance before lockdown, yes vs. no	20/196 (10.2)	63/507 (12.4)	0.80 (0.47–1.36)	0.41		
Public transport use to go to school before lockdown, yes vs. no	18/97 (18.6)	65/598 (10.9)	1.87 (1.05–3.31)	0.03	1.25 (0.64–2.43)	0.51
Persistent SARS-CoV-2 infection in adult, yes vs. no	48/228 (21.1)	37/480 (7.7)	3.14 (1.97–5.00)	<0.001	2.08 (1.24–3.51)	0.006
Rhinovirus/enterovirus infection in child, yes vs. no	26/120 (21.7)	56/581 (9.6)	2.65 (1.60–4.43)	<0.001	2.04 (1.13–3.68)	0.018
Rhinovirus/enterovirus infection in adult, yes vs. no	7/34 (20.6)	77/670 (11.5)	2.00 (0.84–4.74)	0.12		
Antibody detection by LFA, yes vs. no	44/122 (36.1)	40/586 (6.8)	7.70 (4.72–12.56)	<0.001	7.20 (4.27–12.15)	<0.001

Abbreviations: SARS-CoV-2, severe acute respiratory syndrome coronavirus 2; OR, odds ratio; aOR, adjusted odds ratio; CI, confidence interval; LFA, immunochromatographic lateral flow assay. ^a^ Group 1 refers to the variable group mentioned in the first place (i.e., health worker) and Group 2 refers to the variable group mentioned in the second place (i.e., other professions). ^b^ ≥10 events of interest per variable. ^c^ Since January 2020.

## Data Availability

The data presented in this study are available on reasonable request from the corresponding author. The data are not publicly available due to the confidentiality of participants’ information.

## Appendix A

A detailed description of SARS-CoV-2 RNA extraction and amplification procedures is contained in Appendix A.

Nasopharyngeal swabs were introduced into micronic tubes with 750 μL of Zymo DNA/RNA Shield Lysis Buffer™ (Zymo Research, Freiburg, Germany). Each tube was labeled with 2D barcode stickers (Brady Printer i7100) for sample identification. Tubes were transported to the Molecular Microbiology Laboratory of the study site and disinfected on arrival by cleaning the outside with Ethanol 70%. Each sample was aliquoted into 2 sub-samples: one was processed for multiple respiratory virus detection and another was transferred to the laboratory of Centro de Regulación Genómica (Barcelona, Spain) for SARS-CoV-2 RT-PCR testing. Sample surplus was stored at −80 °C in the study setting’s bio bank, which is accredited as a facility integrated into the Spanish National Registry of Biobanks for Biomedical Research, until use for further studies.

SARS-CoV-2 RNA was extracted using the Quick-DNA/RNA Viral Mag Bead kit (Zymo Research) which was fully automated on a Tecan Dream Prep NAP Workstation (Tecan Trading AG, Switzerland). The robot used 360 μL of sample in DNA/RNA Shield buffer and automated all the subsequent steps of magnetic bead sample purification until the elution step, which was performed in 70 μL of RNase-free water. SARS-CoV-2 RT-PCR assays were performed according to CDC-006-00019 CDC/DDID/NCIRD/ Division of Viral Diseases protocol released on 3/30/2020 and available at https://www.fda.gov/media/134922/download, which included the CDC-approved primers and probes for SARS-CoV-2 N1 and N2 genes, as well as RNaseP human gene as internal control. Primers and probes were purchased from IDT integrated technologies (qPCR probes - 2019-nCoV CDC EUA Kit). Five microliters of extracted RNA were mixed with 15 μL of RT-PCR master mix (Luna Universal Probe One-Step RT-qPCR Kit; New England Biolabs; E3006). RT-PCR assays were performed in an Applied Biosystems 7900HT robot with 384 well block equipment. Reverse transcription was carried out for 10 min at 55 °C followed by 45 cycles of quantitative PCR with 95 °C denaturation for 3 s and 30 s annealing/elongation at 64 °C. Output results were transferred to a Laboratory Information Management System that recorded sample barcode information and cycle threshold (Ct) values for N1, N2, and RNAse P. A microbiologist had remote access to the amplification curves so as to analyze and interpret them prior to delivering diagnostic results.

Calibration curves were performed by using ten-fold serial dilutions of reference viral RNA (from 500,000 copies to 1.2 copies/mL per well). Equal volumes of standard and samples were used for RT-PCR amplification of N1 gene and N2 genes. In a base-10 semi-logarithmic graph, two curve graphs were generated by plotting the CT values of N1 or N2 genes on the y-axis and the log of the input amounts on the x-axis. The slope of these standard curves was −3.1 for N1 and −3.2 for N2 and the correlation coefficient was 0.996 in both. Viral load in copies/mL per sample were calculated considering sample and RNA extract elution volumes. N1 gene was regarded as the main gene for viral load calculation except for samples in which only N2 gene was detected.

## References

[1] World Health Organization (2020). Novel Coronavirus (2019-nCoV). Situation Report—1. 21 January 2020.

[2] European Centre for Disease Prevention and Control COVID-19 Situation Update Worldwide, as of Week 24, updated 24 June 2021. from:https://www.ecdc.europa.eu/en/geographical-distribution-2019-ncov-cases.

[3] (2020). Government Decrees State of Emergency to Stop Spread of Coronavirus COVID-19.

[4] Tang Y.-W., Schmitz J.E., Persing D.H., Stratton C.W. (2020). Laboratory diagnosis of COVID-19: Current issues and challenges. J. Clin. Microbiol..

[5] Chen J., Qi T., Liu L., Ling Y., Qian Z., Li T., Li F., Xu Q., Zhang Y., Xu S. (2020). Clinical progression of patients with COVID-19 in Shanghai, China. J. Infect..

[6] Xiao A.T., Tong Y.X., Gao C., Zhu L., Zhang Y.J., Zhang S. (2020). Dynamic profile of RT-PCR findings from 301 COVID-19 patients in Wuhan, China: A descriptive study. J. Clin. Virol..

[7] To K.K., Tsang O.T., Leung W.S., Tam A.R., Wu T.C., Lung D.C., Yip C.C., Cai J.P., Chan J.M., Chik T.S. (2020). Temporal profiles of viral load in posterior oropharyngeal saliva samples and serum antibody responses during infection by SARS-CoV-2: An observational cohort study. Lancet Infect. Dis..

[8] Hazra A., Collison M., Pisano J., Kumar M., Oehler C., Ridgway J.P. (2020). Coinfections with SARS-CoV-2 and other respiratory pathogens. Infect. Control. Hosp. Epidemiol..

[9] Singh V., Upadhyay P., Reddy J., Granger J. (2021). SARS-CoV-2 respiratory co-infections: Incidence of viral and bacterial co-pathogens. Int. J. Infect. Dis..

[10] Nowak M.D., Sordillo E.M., Gitman M.R., PanizMondolfi A.E. (2020). Coinfection in SARS-CoV-2 infected patients: Where are influenza virus and rhinovirus/enterovirus?. J. Med. Virol..

[11] Kim D., Quinn J., Pinsky B., Shah N.H., Brown I. (2020). Rates of co-infection between SARS-CoV-2 and other respiratory pathogens. JAMA.

[12] Xia W., Shao J., Guo Y., Peng X., Li Z., Hu D. (2020). Clinical and CT features in pediatric patients with COVID-19 infection: Different points from adults. Pediatr. Pulmonol..

[13] Pinky L., Dobrovolny H.M. (2020). SARS-CoV-2 coinfections: Could influenza and the common cold be beneficial?. J. Med. Virol..

[14] Wee L.E., Ko K.K.K., Ho W.Q., Kwek G.T.C., Tan T.T., Wijaya L. (2020). Community-acquired viral respiratory infections amongst hospitalized inpatients during a COVID-19 outbreak in Singapore: Co-infection and clinical outcomes. J. Clin. Virol..

[15] Zhu X., Ge Y., Wu T., Zhao K., Chen Y., Wu B., Zhu F., Zhu B., Cui L. (2020). Co-infection with respiratory pathogens among COVID-2019 cases. Virus Res..

[16] Brotons P., Launes C., Buetas E., Fumado V., Henares D., de Sevilla M.F., Redin A., Fuente-Soro L., Cuadras D., Mele M. (2020). Susceptibility to SARS-CoV-2 infection among children and adults: A seroprevalence study of family households in the Barcelona metropolitan region, Spain. Clin. Infect. Dis..

[17] Zhou F., Yu T., Du R., Fan G., Liu Y., Liu Z., Xiang J., Wang Y., Song B., GU X. (2020). Clinical course and risk factors for mortality of adult inpatients with COVID-19 in Wuhan, China: A retrospective cohort study. Lancet.

[18] Xiao A.T., Tong Y.X., Zhang S. (2020). False negative of RT-PCR and prolonged nucleic acid conversion in COVID-19: Rather than recurrence. J. Med. Virol..

[19] Yuan J., Kou S., Liang Y., Zeng J., Pan Y., Liu L. (2020). Polymerase chain reaction assays reverted to positive in 25 discharged patients with COVID-19. Clin. Infect. Dis..

[20] Sohn Y., Jeong S.J., Chung W.S., Hyun J.H., Baek Y.J., Cho Y., Kim J.H., Ahn J.Y., Choi J.Y., Yeom J.S. (2020). Assessing viral shedding and infectivity of asymptomatic or mildly symptomatic patients with COVID-19 in a later phase. J. Clin. Med..

[21] Laferl H., Kelani H., Seitz T., Holzer B., Zimpernik I., Steinrigl A., Schmoll F., Wenisch C., Allerberger F. (2021). An approach to lifting self-isolation for health care workers with prolonged shedding of SARS-CoV-2 RNA. Infection.

[22] Bullard J., Dust K., Funk D., Strong J.E., Alexander D., Garnett L., Boodman C., Bello A., Hedley A., Schiffman Z. (2020). Predicting infectious severe acute respiratory syndrome coronavirus 2 from diagnostic samples. Clin. Infect. Dis..

[23] La Scola B., Le Bideau M., Andreani J., Hoang V.T., Grimaldier C., Colson P., Gautret P., Raoult D. (2020). Viral RNA load as determined by cell culture as a management tool for discharge of SARS-CoV-2 patients from infectious disease wards. Eur. J. Clin. Microbiol. Infect. Dis..

[24] Arons M.M., Hatfield K.M., Reddy S.C., Kimball A., James A., Jacobs J.R., Taylor J., Spicer K., Bardossy A.C., Oakley L.P. (2020). Presymptomatic SARS-CoV-2 infections and transmission in a skilled nursing facility. N. Engl. J. Med..

[25] Hoffmann M., Kleine-Weber H., Schroeder S., Krüger N., Herrler T., Erichsen S., Schiergens T.S., Herrler G., Wu N.H., Nitsche A. (2020). SARS-CoV-2 cell entry depends on ACE2 and TMPRSS2 and is blocked by a clinically proven protease inhibitor. Cell.

[26] Murphy R.C., Lai Y., Barrow K.A., Hamerman J.A., Lacy-Hulbert A., Piliponsky A.M., Ziegler S.F., Altemeier W.A., Debley J.S., Gharib S.A. (2020). Effects of asthma and human rhinovirus A16 on the expression of SARS-CoV-2 entry factors in human airway epithelium. Am. J. Respir. Cell Mol. Biol..

[27] Kuba K., Imai Y., Rao S., Gao H., Guo F., Guan B., Huan Y., Yang P., Zhang Y., Deng W. (2005). A crucial role of angiotensin converting enzyme 2 (ACE2) in SARS coronavirus-induced lung injury. Nat. Med..

[28] Imai Y., Kuba K., Rao S., Huan Y., Guo F., Guan B., Yang P., Sarao R., Wada T., Leong-Poi H. (2005). Angiotensin converting enzyme 2 protects from severe acute lung failure. Nature.

[29] Lauer S.A., Grantz K.H., Bi Q., Jones F.K., Zheng Q., Meredith H.R., Azman A.S., Reich N.G., Lessler J. (2020). The incubation period of coronavirus disease 2019 (COVID-19) from publicly reported confirmed cases: Estimation and application. Ann. Intern. Med..

